# Embedding effective depression care: using theory for primary care organisational and systems change

**DOI:** 10.1186/1748-5908-5-62

**Published:** 2010-08-06

**Authors:** Jane M Gunn, Victoria J Palmer, Christopher F Dowrick, Helen E Herrman, Frances E Griffiths, Renata Kokanovic, Grant A Blashki, Kelsey L Hegarty, Caroline L Johnson, Maria Potiriadis, Carl R May

**Affiliations:** 1 Primary Care Research Unit, The Department of General Practice, School of Medicine, The University of Melbourne, Australia; 2 Department of Primary Care, School of Population, Community and Behavioural Sciences, University of Liverpool, Liverpool, UK; 3 Centre for Youth Mental Health, The University of Melbourne, Australia; 4 Centre for Primary Health Care Studies, Warwick Medical School, University of Warwick, UK; 5 Department of Sociology, School of Political and Social Enquiry, Monash University, Australia; 6 Nossal Institute for Global Health, The University of Melbourne, Australia; 7 Institute of Health and Society, Newcastle University, UK

## Abstract

**Background:**

Depression and related disorders represent a significant part of general practitioners (GPs) daily work. Implementing the evidence about what works for depression care into routine practice presents a challenge for researchers and service designers. The emerging consensus is that the transfer of efficacious interventions into routine practice is strongly linked to how well the interventions are based upon theory and take into account the contextual factors of the setting into which they are to be transferred. We set out to develop a conceptual framework to guide change and the implementation of best practice depression care in the primary care setting.

**Methods:**

We used a mixed method, observational approach to gather data about routine depression care in a range of primary care settings via: audit of electronic health records; observation of routine clinical care; and structured, facilitated whole of organisation meetings. Audit data were summarised using simple descriptive statistics. Observational data were collected using field notes. Organisational meetings were audio taped and transcribed. All the data sets were grouped, by organisation, and considered as a whole case. Normalisation Process Theory (NPT) was identified as an analytical theory to guide the conceptual framework development.

**Results:**

Five privately owned primary care organisations (general practices) and one community health centre took part over the course of 18 months. We successfully developed a conceptual framework for implementing an effective model of depression care based on the four constructs of NPT: coherence, which proposes that depression work requires the conceptualisation of boundaries of who is depressed and who is not depressed and techniques for dealing with diffuseness; cognitive participation, which proposes that depression work requires engagement with a shared set of techniques that deal with depression as a health problem; collective action, which proposes that agreement is reached about how care is organised; and reflexive monitoring, which proposes that depression work requires agreement about how depression work will be monitored at the patient and practice level. We describe how these constructs can be used to guide the design and implementation of effective depression care in a way that can take account of contextual differences.

**Conclusions:**

Ideas about what is required for an effective model and system of depression care in primary care need to be accompanied by theoretically informed frameworks that consider how these can be implemented. The conceptual framework we have presented can be used to guide organisational and system change to develop common language around each construct between policy makers, service users, professionals, and researchers. This shared understanding across groups is fundamental to the effective implementation of change in primary care for depression.

## Background

Depression and related disorders represent a significant part of general practitioners (GPs) daily work [1,2]. Internationally, governments and service providers are grappling with how to improve the delivery and systems for depression care to reduce the personal and financial burden on the health care system and society. Improving depression care is complicated by difficulties researchers and policy makers face in terms of the transfer and implementation of the evidence about what works into routine practice [2,3]. For example, the 'collaborative care model' for depression care, originating in the USA, has shown promise for improving patient health outcomes for depression [4-6], but there is uncertainty as to whether this model of care will effectively translate to other health care systems and routine embedding within the setting in which it was developed has not yet occurred [7]. Locally specific trials are underway in the UK [8,9], the Netherlands [10], and India [11] that will provide further insight into this question. There is also growing awareness that complex interventions, such as 'collaborative care,' require careful attention to theory, process, and context [12,13] to maximise their effectiveness and to facilitate the likelihood of transfer into routine clinical care. The emerging consensus is that the transfer of efficacious interventions into routine practice is strongly linked to how well the interventions take into account the contextual factors of the setting into which they are to be transferred [14].

The focus on the importance of understanding, and taking into account, contextual factors has informed the revision of the 2008 Medical Research Council (MRC) guidance for the evaluation of complex interventions [12]. There is a call for greater emphasis on the use of theory to inform the design of interventions and for more time to be spent on piloting and refining an intervention prior to evaluating effectiveness. To date, intervention design has experienced somewhat of a theoretical vacuum [15]; depression interventions are no exception. The next challenge is to ensure that the implemented interventions are sustainable. This has led to a call for so-called 'self-improving health systems,' which are built upon a culture of continuous learning, reflection, and service improvement [16,17].

In view of this, we began the re-order (re-organising care for depression and related disorders in the Australian primary care setting) project. Re-order was undertaken over three years and sought to explore, in-depth, contextual factors impacting on depression care in order to define what is required for an effective model of depression care and how that model of care might be implemented. Table 1 presents a summary of our previously published research, which involved a wide stakeholder consultation to gather the views of patients and community members about what is required for depression care [18]. Based on extensive consultations with over 500 primary care patients and 300 community members from non-government, government, academic, and other health services, re-order identified a conceptual design for an effective model and system of depression care. The design is based on three domains of care: the relational, the competency, and systems domains [18]. The aim of this paper is to report our in-depth work with six primary care organisations to identify the components of an effective model of depression care. We present this work as a conceptual framework to guide how to implement organisational and systems change in mental health care reform in primary care.

**Table 1:** Summary of stakeholder informed conceptual design of an effective model and system of depression care

Domain	Criteria
Requirements in the **Relational **Domain	Stakeholders want to be 'listened to,' 'understood,' 'empathised with,' 'supported,' 'reassured,' and 'encouraged' by care providers (particularly GPs), receive depression care that is 'holistic,' 'tailored to the individual,' and 'involves the patient in planning.'
Requirements in the **Competency **Domain	Stakeholders want 'competent and thorough diagnosis and management,' 'assessment for severity and suicide risk,' 'appropriate and timely referrals,' 'incorporation of social factors,' 'monitoring and follow up,' 'education about depression,' and 'prescription and management of medication.'
Requirements in the **Systems **Domain	Stakeholders want 'funding for longer consultations and follow-up,' 'systems to enable monitoring,' 'timely referral through a range of treatment options,' 'the integration of primary care and other providers,' and 'professional support to general practice.'
How can the effectiveness of the **Relational **Domain be assessed?	'Measuring patient satisfaction,' 'surveying patients, carers, GPs and consumer groups,' and 'monitoring patient recovery.'
How can the effectiveness of the **Competency **Domain be assessed?	'Measuring whether there is less reliance on medication and a medical model,' 'monitoring recovery and diagnosis rates,' 'monitoring patients capacity to function physically, socially, and in the community,' and 'developing appropriate prescribing.'
How can the effectiveness of the **Systems **Domain be assessed?	'Measuring for 'increases in referral options and services in regional areas,' 'patient satisfaction,' 'access and affordability of services,' 'monitoring referrals made by GPs,' 'monitoring the duration and quality of follow up,' 'monitoring the number of patients seeking help,' and 'monitoring collaboration.'

## Methods

To explore the context of primary care and the way it responds to people experiencing depression, our approach was informed by the view that primary care is a complex adaptive system (CAS) [19,20]. Such systems are said to consist of different members and components that are dynamic, interactive, and dependent. These systems are adaptive with the capacity to change and to self-organise; they have shadow systems operating in daily work; they have emergent properties that are more than the sum of individual parts, and show initial conditions that can markedly influence what happens in practice [21]. We sought to collect data to understand all of these elements at work in a number of primary care organisations. To identify the components of an effective system for depression care, we first sought to understand how depression care was functioning in each organisation. To facilitate this, we used a method informed by the principles of participatory action research (PAR) [22] and utilised a mix of quantitative and qualitative methods as outlined below.

Approval was sought and gained from the Human Research Ethics Committee at The University of Melbourne HREC Approval No. 120406.

### Sample

**Figure 1:**
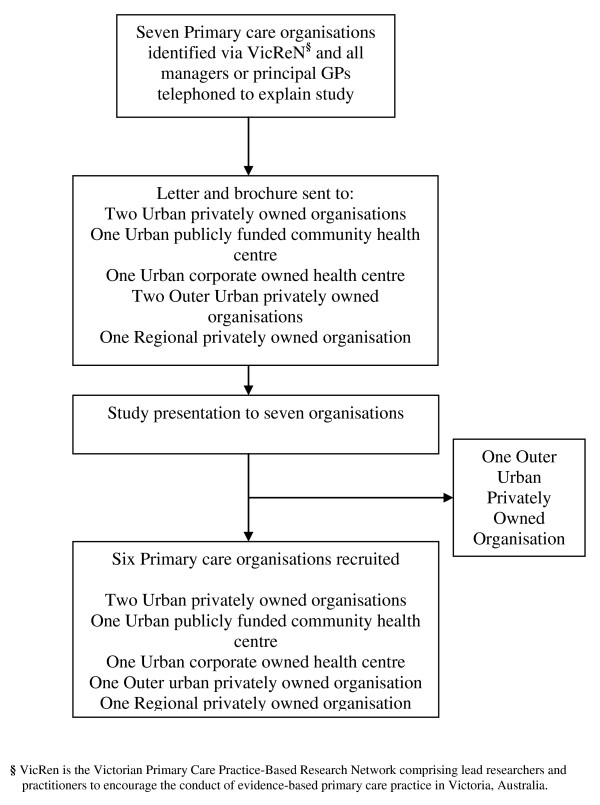
**Recruitment flowchart for re-order**.

Organisations were purposefully sampled from urban, outer urban, and regional locations of Victoria and Tasmania. Purposeful sampling is a common method of recruitment in qualitative research and sites are selected to provide information rich cases that reveal in-depth understanding rather than empirical generalisations [23]. As re-order sought to identify a model and system of depression care informed by currently available best practice, we sampled from the Victoria Practice-Based Research Network (VicReN). Member organisations of VicReN were deemed to be the most likely candidates to illustrate best practices (although there are many examples of excellent care delivered in a variety of settings). The sample size was intentionally small due to the extensive data collection process and high level of participation required from practices. Figure 1 illustrates the recruitment process undertaken.

Seven eligible primary care organisations were identified. Each had from two up to ten or more GPs working within them plus other professionals (receptionists, practice nurses, dieticians, diabetic nurse educators, psychologists, and social workers). Five organisations were privately owned by principal GPs, one was a corporate owned health centre, and one was a publicly funded community health centre. A researcher telephoned the manager or principal GP to explain the study and sent a formal letter of invitation and an information brochure to practices. An organisational meeting was schedule for all staff including receptionists, practice nurses, GPs and any other health professionals employed. A 30-minute presentation was delivered to all seven organisations. The research team outlined the study aims, available policy, and research evidence on depression care and the data collection processes. Organisations that agreed to participate were paid $5,000 (AU) remuneration for their time taken to facilitate data collection and to attend meetings.

### Data collection

**Figure 2:**
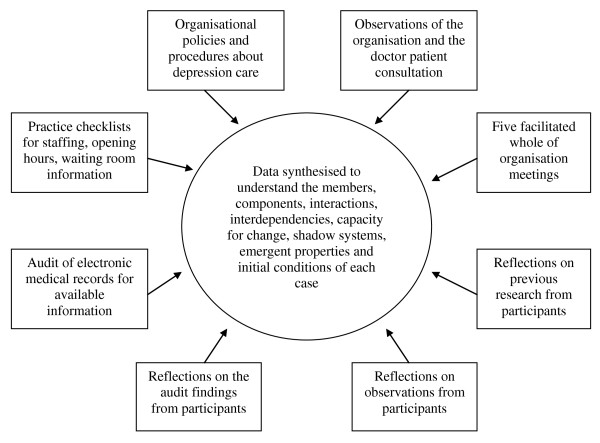
**Data collection methods for re-order**.

Data collection was conducted over 18 months (2007 to 2008). Combinations of qualitative and quantitative data collection methods were used to understand each organisation as a CAS; these methods are illustrated in Figure 2. The research methods were informed by previous studies that had sought to describe family practice in the US through the lens of complexity theory [24-27].

### Quantitative methods

The audit method [28] was employed to identify readily available information about the numbers of adult patients in the previous 12-month period who had an existing diagnosis of depression, and/or were taking antidepressant medications and how often they attended clinics. The audits were facilitated by a trained research assistant (MP) who assisted key staff to search medical records. MP also completed practice checklists to document the opening hours of practices, number of full-time equivalent staff, information readily available to patients in waiting rooms, and to produce an individual floor plan of each organisation and its physical layout.

### Qualitative methods

Available documents on depression care including policy and procedures were collected from organisations to inform our understanding of the context in which we were observing practice. A graduate anthropologist (BK) visited practices each week for up to eight months to conduct observations [26]. Field notes were written by the observer detailing their perspective on commonly experienced behaviours, routines, events, and the setting [29]. In addition to this, all staff participated in monthly meetings that were audio recorded and professionally transcribed. Meetings included receptionists, practice nurses, GPs, and other health professionals; all participant names and organisations were de-identified and pseudonyms were allocated. Transcripts were checked for quality assurance by listening to selections of audio files and cross checking with transcripts for accuracy (VP).

A non-medically trained person facilitated the meetings (VP) using PAR methods to engage participants in a process of observation, reflection, and discussion [22]. Using PAR approaches enabled us to develop understanding from the bottom up about the context and processes used for depression care within each organisation. Structured activities were used in the meetings, which included: staff identifying their perceived strengths, weaknesses, opportunities, and challenges for depression care; their individual views on depression and the system of depression care; discussing the audit findings; providing feedback on previously gathered data on what is required for an effective model and system of depression care; reflecting back the observations of the observer; and identifying possible areas of change from the organisational level to improve depression care. Data collected from meetings were used to inform the development of the conceptual framework for embedding effective depression care in the primary care setting.

### Data analysis

**Figure 3:**
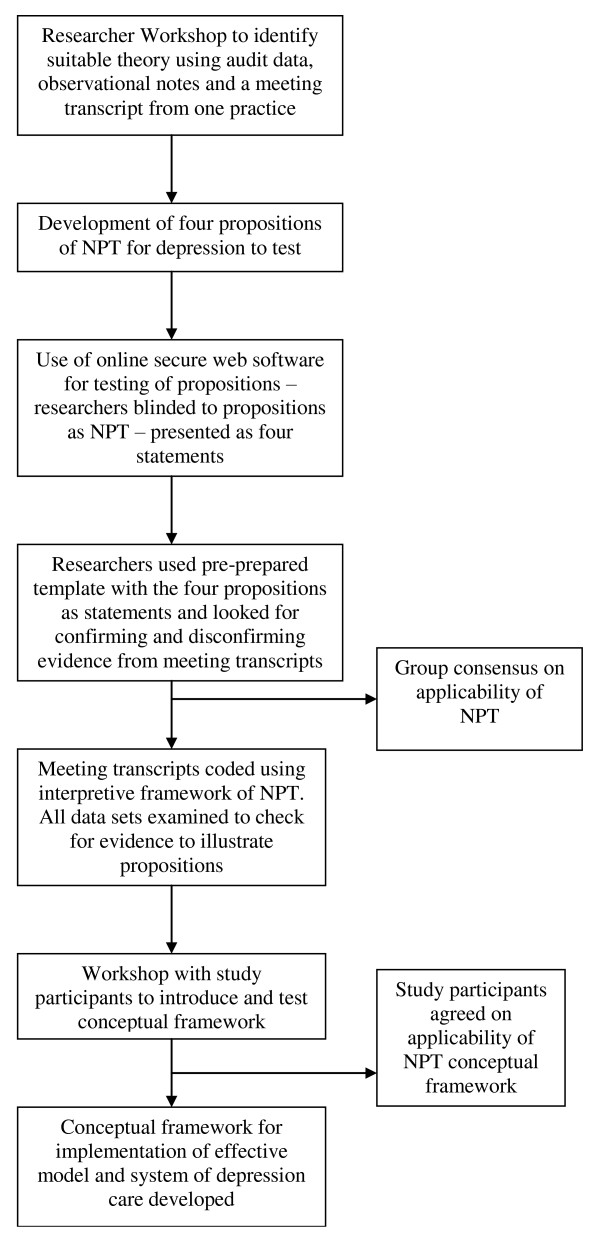
**Theory-building process for conceptual framework**.

All data sets from each organisation were combined and considered as a whole case. Although the aim of the re-order project was to identify the components of an effective model of depression care, data revealed diffuse processes and systems of practice for depression care. While components of depression care were evident, cases indicated that more theoretical consideration was required about how to facilitate organisational and system change to implement effective models of depression care. As a result, the study team decided that Normalisation Process Theory (NPT, see below) could provide an analytical theory to develop a conceptual framework to guide the implementation of an effective model and system of depression care. The process of identifying and testing NPT suitability for this task is outlined in Figure 3.

### NPT as an analytical framework

We selected NPT to guide our analysis as it provides an 'explanatory framework for investigating the routine embedding of material practices in social contexts' [30]. NPT is based upon four interactive constructs termed 'coherence,' 'cognitive participation,' 'collective action,' and 'reflexive monitoring.'[31-33]. NPT postulates that in order to become a routine, practice work has to be done to define and organise the objects of a practice (coherence), participants have to enrol in a work practice (cognitive participation), work has to be undertaken to define and organise the enacting of a practice (collective action), and work has to be done to define and organise the knowledge upon which appraisal of a practice is founded (reflexive monitoring). The starting point of NPT is 'what is the work?' [30].

Our first step was to explore how NPT could be applied to depression care. JG, VP, and CM initially met to develop four propositions for depression care that corresponded with each construct (see Table 2). Table 2 also outlines the set of questions JG and VP developed for each proposition to guide the analysis of meeting transcripts.

**Table 2:** Interpretive framework of NPT developed and applied for analysis

Propositions Developed and Tested	Corresponding Constructs May and Finch	Our interpretation of the constructs to guide data analysis
Depression work requires conceptualisation of boundaries (who is depressed/who is not depressed). Depression work requires techniques for dealing with diffuseness.	*Coherence* *(Do people know what the work is?)*	How do participants conceptualise boundaries around depression care work? Is there evidence that depression is viewed as a diffuse problem? What is the meaning attributed to depression and depression work. How is depression work specified and differentiated? What practices define depression work? Are these practices more than a set of acts?
Depression work requires engagement with a shared set of techniques that deal with depression as a health problem.	*Cognitive Participation* *(Do people join in to depression work?)*	How do participants engage with, initiate and enrol in depression work? How is depression work legitimated? What norms and conventions of practices exist around depression care? Is there evidence of joining and buying in to depression work?
Depression work requires agreement about how care is organised-who is required to deliver care, and their structural and human interactions.	*Collective Action* *(Skill-Set Workability & Interactional Workability)* *(How do people do the work?)*	**Skill Set Workability: **Examples of external rules (formal and informal) that govern depression work and the relationship between these and behaviours. (Policies for example). Examples of the organisation of the work - divisions of labour; who does what and how it is performed? **Contextual Integration: **How is work resourced? Where is the power? Is there formal or informal agreement about the value of work? **Interactional Workability: **How is the work conducted? What are the informal rules that govern this work? Examples of cooperation to do the work. Examples of goals set for the work. Examples of the meaning given to the work. **Relational Integration: **How is the work dispersed?
Depression work requires the ongoing assessment of how depression care is done.	*Reflexive Monitoring* *(How do we know that the work is happening?)*	How do people review and reflect upon depression work? How is depression care monitored?

The propositions were tested for adequacy by members of the research team (CD, FG, HH, KH, RK, CJ), not involved in the analysis to date. Testing occurred using a secure web-based file sharing system. Members of the research team were provided with an analysis template that had each proposition listed out as a statement. There was no reference or indication of the relationship of the statements to NPT constructs. Members of the research team read selections of meeting transcripts and observer notes to find examples that confirmed or disconfirmed each statement. This approach worked well with investigators participating in the task and agreeing that the four propositions could cover the issues spoken about within the transcripts.

JG and VP applied the NPT constructs and propositions to each meeting transcript. All data from transcripts were coded to a particular proposition until data saturation occurred. We checked audit data and observational notes for examples that supported the four propositions also. Our final step was to present our ideas for the conceptual framework back to representatives from each organisation at a workshop on completion of the study. At this final workshop, we observed the participants working with the proposed framework as they identified examples of what is required for each construct (coherence, cognitive participation, collective action, and reflexive monitoring) and planned how to implement best practice depression care.

## Results

Six primary care organisations were recruited as shown in Table 3. Organisations varied in ownership and size. Five were privately owned (four were owned by principal GPs and one was a corporate owned health centre) one was a public funded community health centre. Organisations were located in urban (n = 4), outer urban (n = 1), and a regional centre of Tasmania, Australia (n = 1). Each organisation had other health care staff and receptionists employed, and many had co-located allied health and psychologists within their practice. The second outer urban practice declined to participate due to heavy teaching commitments.

**Table 3:** Participating Organisations and Characteristics

	Practice (n=number of participating GPs at commencement)					
Organisational Characteristics	Eastvale (n = 5)	Gibson (n = 1)	Frank (n = 4)	Southville (n = 7)	Coopers (n = 7)	West Sanders (n = 9)
**Funding Structure**						
Privately owned primary care sites	**Y**	**Y**	**Y**	**Y**		
Corporatised primary care site						**Y**
Publicly funded community health centre					**Y**	
**Location**						
Urban	**Y**	**Y**			**Y**	**Y**
Outer Urban				**Y**		
Regional			**Y**			
**Personnel employed in the practice (in total)**						
GP(s)	6	2	4	8	8	14
Practice nurse(s)	2	3	2	4	2	3
Registrar(s)	1	0	0	0	0	1
Psychologist(s)	1	1	0	2	1	1
Practice manager(s)	1	0	1	1	1	1
Receptionist(s)	6	2	3	10	5	7
Other	2	0	2	7	10	0

While re-order sought a whole of organisation approach, participation varied as illustrated in Table 4. Frank had 8/12 (66.7%) participants, Gibson 5/8 (62.5%), Eastvale 10/19 (52.6%), Coopers 11/27 (40.7%); Southville 12/32 (37.5%), and West Sanders 9/27 (33.3%). The larger sized organisations of Southville and West Sanders had lower participation rates due to numbers of reception staff who did not participate. Other participation rates were affected by staff not being rostered on the day meetings were held, annual leave arrangements, and the part-time nature of many staff. These factors affected attendance rates at monthly meetings. Although the research team suggested that all staff participate, we were not aware of any co-located psychologists being invited. There were 55/123 (44.7%) professional participants across all organisations. Participation from professional groups consisted of 28/42 (66.7%) GPs, 9/16 (56.3%) practice nurses, 3/5 (60%) managers, 3/33 (9.1%) receptionists, 0/6 (0%) co-located psychologists, 11/21 (52.4%) other professionals (a mix of social workers, dieticians, interpreters, and other practice professionals).

**Table 4:** Staff Participation

Study organisation (n=total staff)	Participants (N = 55)						
	GP^‡^	PM^†^	PN^±^	Rec*	Other^	Total	Participation (%)
**Eastvale (n = 19)**	4	1	2	3	0	10	(52.6)
**Gibson (n = 8)**	1	0	3	1	0	5	(62.5)
**Frank (n = 12)**	3	1	0	0	4	8	(66.7)
**Southville (n = 32)**	7	0	3	0	2	12	(37.5)
**Coopers (n = 27)**	4	1	1	0	5	11	(40.7)
**West Sanders (n = 27)**	9	0	0	0	0	9	(33.3)
**TOTAL**	**28**	**3**	**9**	**4**	**11**	**55**	

‡GP = General Practitioner, †PM = Practice Manager, ± PN = Practice Nurse, *Rec = Receptionist, ^Other = includes other practice health professionals

### Using NPT to develop a conceptual framework

Table 5 shows a selection of examples identified from transcripts to illustrate each proposition and construct. These examples informed the development of a conceptual framework for how to implement and embed an effective model and system of depression.

**Table 5:** Participant views informing the conceptual framework

Domain	Participant Views
**COHERENCE**	**The meaning of depression**
**Developing a shared understanding of what constitutes depression and depression work**	...In the end a lot of the so-called 'depression' that we see is related to practical issues like, they haven't got a job or they're caring for five children and a sick grandma, all of those sorts of things...they're not sitting there with existential angst wondering about the meaning of life. It's because of practical issues they're so-called 'depressed' in many cases (GP Coopers Road Practice Meeting 2: 12). ...I think so often they're so deeply meshed, the physical, the emotional and the psychological, that as soon as you start impact on one, you end up impacting on the other...[psychologists] are not sitting there thinking, 'gosh is this the manifestation of heart disease'? So, GPs have got a step before then. I don't see that these are two separate things that are warring with each other -- the psychological versus the physical. It's just part of the melting pot, the mess really (GP Coopers Road Practice Meeting 3: 5). ...If someone who came to seem me as initially a first port of call, I would probably try to work that through. The next level you've got in my mind is, that, I'm starting to realise that the next level of patients are in that 'grey zone,' they've got mood disorders, they have all sorts of issues with work, family, illness and what have you. They're not quite classically, fully depressed by a DSM-IV criteria, but they are in what some people now seem to be calling a disregulated zone. They are not quite fully depressed, but they're not quite right (GP Eastvale Practice Meeting 3: 13). **Diffuse boundaries** ...Diagnosis Management is so hard. Do you have to define it and say this is depression, this is anxiety. I don't think that you can (GP West Sanders Practice Meeting 5: 13). **The meaning of depression work** ...What I will often do, is, if I'm seeing somebody and I think, 'well, is this masked depression presenting'? I'll just put 'query depression, investigate next attendance.' So the next time that they come in I take the opportunity to then take it further and look at it....I think that happens with depression as there's so many different gradients (GP Franklin Street Practice Meeting 4: 13). ...I think sometimes though, if you're focusing on a psychological problem you have to be careful that you don't actually miss the very obviously physical problem, that there is some pathology going on that you need to try and treat with medication. Sometimes, it's finding that balance (GP Coopers Road Practice Meeting 2: 5). ...I think what would probably be the biggest concern, from our perspective...is because you know you're going to miss -- at the end of the day, you're going to miss things -- and you're going to miss things in depression, or going to miss it in heart disease or stroke or all of those things. Consequently you're constantly aware that the next patient who comes in could have a problem that, if you miss, could have a profound effect on the rest of their lives. That happens every 15 minutes (GP Franklin Street Practice Meeting 2: 21).

**COGNITIVE PARTICIPATION**	**Agreement on techniques**
**Agreement and engagement with a shared set of techniques that deal with depression as a health problem**.	...Look, I think with depression it is a bit of give and take. I think when you are seeing a patient who is depressed you often ask, 'well, what are your expectations? You've come to see me regarding depression, what are your thoughts and how can I offer assistance'? It's not just a matter of saying you're depressed, this is what you're going to take and, you know, it will go away. I mean obviously it's an interaction and the whole idea of the doctor patient interaction is to actually work out what the expectations are with the patient and how best to manage that. If it means further referrals and psychological interventions, if it means just listening, if it means regular reviews, finding more time, I mean you work that out with the patient (GP Southville Practice Meeting 1: 19). ...You know, you tell [patients] what to do [for hypertension] and they go, 'good.' For depression, they go, 'no I'm not taking antidepressants.' You know, they have much more fixed ideas, and for various reasons. So, there's a lot more finding out where they're at, and then negotiating your way through than for a lot of straightforward medical illness (GP West Sanders Practice Meeting 1: 23). **Engagement with shared techniques (patients included)** ...Look, someone was in yesterday who I think has been depressed for ages and was talking about this and I said to her, 'look, you are really depressed. We need to talk about this.' She knew that something was not right, but she really didn't want to go there...that sort of stuff happens quite often (GP Gibson Practice Meeting 3: 2). ...What do you do if you make a diagnosis but the patient refuses to accept it? I had two patients...one, she just had this terrible half a dozen years, the business went bankrupt and her marriage broke up and she's changed jobs about four times. Her dad died, her mother died when she was young and she's no longer speaking to her brother because of the fights about the will and because there was the new wife who had the fights about the will and [the patient] felt that she was left to do the fighting. Yet, she's says that she's not depressed because people in her family are not depressed...So what do you write in her notes? If I say to this patient, 'I think that you're depressed,' and they say, 'no, I'm not,' then do you put it in their notes? (GP Franklin Street Practice Meeting 4: 7-8). **Legitimacy of depression as a health problem** ...I wouldn't have thought we had that many patients with depression presenting previous to the government funding coming in [for structured mental health plans]...because sometimes I think that maybe they are not really depressed but because it is rebated they are coming in? (Receptionist Gibson Practice Meeting 2: 15).

**COLLECTIVE ACTION**	**Skill set workability**
**Agreement about how care is organised. Who is required to deliver care, and their structural and human interactions**.	...A couple of patients come to mind because there has been a combination of assessing the depression, then there was housing, then there was visa, then there was parenting and, you know, there were services just flying everywhere and I was trying to figure out how to combine them...It was Monday you go to her, Tuesday you go there and Wednesday you go there. So I found that a bit overwhelming in terms of how to pull that together and even to get them to see the people they needed (GP Coopers Road Practice Meeting 4: 21). ...I mean, I find it very hard to get your patients booked in with private psychiatrists, especially as a lot of psychiatrists have got closed books (GP Southville Practice Meeting 4: 18). ...I saw in this general practice, this mental health nurse was actually facilitating the care in a way that took a lot of the arduousness out of if for the GP and in doing that she did a bit of low grade kind of counseling at the same time as doing the process (GP West Sanders Practice Meeting 5: 6). ...I don't think it's appropriate for practice nurses to do depression care, it's a three year course (Practice Nurse Southville Practice Meeting 3: 13). **Contextual integration** ...I don't want to leave the consulting room to go out and get one of those [depression] brochures and then walk back in and give it to the patient (GP Southville Practice Meeting 4: 3). ...The trouble is that importing portable document files (PDFs) into our electronic medical record system is an exercise in intermittent frustrations because sometimes they stay and sometimes they don't. We've tried to do it before (GP Southville Practice Meeting 4: 5). ...The other thing that would help toward a model of depression care is having a more thorough database for referrals. I think it's quite difficult sometimes to assess or to know which psychologists have experience or expertise in particular areas. The same even with psychiatrists. Sometimes it feels like you're just sort of sending patients off a bit blindly and hoping it works out (GP West Sanders Practice Meeting 5: 9). **Interactional workability** ...With the resources, I don't think that I'd be giving anything out unless really Meredith (GP) said you could give them such and such because I wouldn't know what to give out for the type of condition the patient has got (Practice Nurse Gibson Street Practice Meeting 4: 19). **Relational integration** ...I guess just in terms of the mental health care nurse, I am not clear which part of it I'd be happy for someone else to do (GP West Sanders Practice Meeting 5:7). ...I think, from my point of view it is recognition. I certainly don't know of patients that have depression. How am I to know? How is that going to be flagged to me, that this particular person is somebody that I have to spend that extra three to four minutes with....so that is my concern (Receptionist Eastvale Practice Meeting 3: 14). ...The thing that I find is that I don't think that I'm skilled enough to do the counseling that psychologists can do. I mean they really are doing this day in and day out - we're actually doing a lot of other things. I mean we're diagnosing a lot of other different illnesses, treating a lot of different illnesses...Even if we did have more time, I don't think GPs, the majority of us are trained enough to be able to input the strategies that psychologists can (GP Southville Practice Meeting 2: 14).

**REFLEXIVE MONITORING**	**Monitoring for effective depression care**
**Depression work requires the ongoing assessment of how depression care is done**.	...A lot of psychologists don't have any time or really much to do with doctors because the ones that, even the ones that we've had long term close liaison with, it's been a battle for them to get their acts together and prepare letters...it's something professionally that they've never done - they've seen themselves as quite separate (GP Eastvale Practice Meeting 3: 10). ...For monitoring quantitative auditing could help and Balint groups and some sort of organised support mechanisms for GPs (GP Coopers Road Evaluation Meeting 1: 1). ...What are the measures? Is the care - what the patient wants or what the evidence would suggest would help them? (GP Franklin Street Evaluation Meeting 1:1). ...Always a follow-up visit. It is amazing that follow up visit. I reckon almost 50% feel - they've had the blood tests, they've been understood, and they're actually able to move on from there, with very little extra support (GP West Sanders Practice Meeting 2: 13).

**Figure 4:**
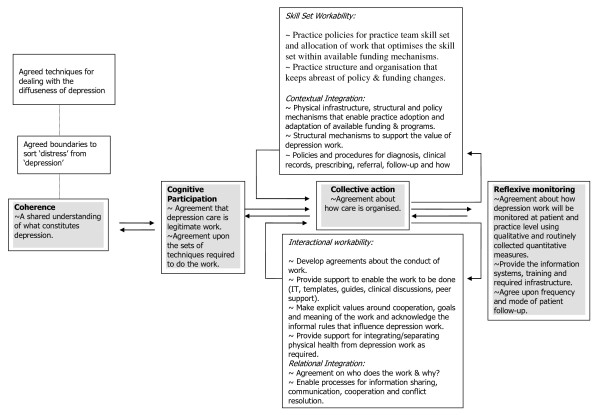
**A conceptual framework to implement an effective model and system of depression care**.

While the constructs are presented in a sequence in Figure 4, they should be thought of as operating concurrently in practice; the system will only function seamlessly if all are present and attended to. Our starting point for implementing an effective model of depression care is based on the construct of coherence and the proposition that depression work requires the conceptualisation of boundaries of who is depressed and who is not depressed, and techniques for dealing with diffuseness. To facilitate the routine adoption of an effective model and system of care, all actors need to have a shared understanding of what depression and depression work means.

During the structured activities conducted in meetings, staff from receptionists, GPs to practice nurses demonstrated a variety of meanings for depression. Descriptions of depression care as 'a maze,' 'complex,' 'interconnected,' 'grey or uncertain,' 'not black and white,' 'multi-factorial,' 'a journey,' 'confusing,' 'diffuse and discursive,' 'amorphous,' 'mysterious,' 'complicated,' and 'strongly embedded' were commonly used. GPs, as Table 5 highlights, saw depression as difficult to categorise because of the interrelationship with social and practical issues for patients, and the inseparable nature of many physical, emotional, and psychological issues.

GPs discussed the challenges of sorting out distress from depression and in particular not missing or overlooking physical problems. Two distinct practice styles appeared to be in operation -- clinicians tended to be either integrators (seeing physical and mental health as inextricably linked dealing with both within a single consultation) or separators (those who tended to deal with physical health and mental health separately). This illustrated that depression work is not neat and easily articulated. As Table 5 also shows, GPs were aware of the diagnostic criteria of Major Depressive Disorder according to the Diagnostic and Statistical Manual for Mental Disorders DSM-IV [34], but they questioned the usefulness and applicability of these criteria to the general practice setting. Patients were described as presenting in a 'grey zone' and GPs outlined that their work was to explore the set of presenting symptoms or problems using clinical and communication skills. They placed this in the context of the patient with their current and prior knowledge of the person and their social situation.

Our data analysis showed that to date there is not a shared understanding about what constitutes depression and depression work in the primary care setting. The importance of developing this is outlined in coherence in Figure 4. This understanding needs to emerge in conjunction with construct two cognitive participation and the accompanying proposition that depression work requires engagement with a shared set of techniques that deal with depression as a health problem. Construct two focuses attention on the need to get practice staff to actively engage and 'join-in' with depression work. More than this, however, is a need to acknowledge the role of the patient and important carers, family members, and friends in cognitively participating in depression work and the sets of techniques used as a legitimate health problem.

Table 5 illustrates the current techniques for dealing with depression as a health problem fall into two main areas of discussion: 'diagnostic' techniques and 'management or treatment' techniques. Validated or structured symptom checklist tools to assist with diagnosis were spontaneously mentioned within some groups, usually in the context of not adding much to what was already known by the doctor. Rarely, the option of a second opinion was mentioned as a useful diagnostic tool, as was a 'trial of treatment.' Negotiating expectations with the patient was commonly outlined as was referral, psychological intervention, listening, reviewing, and finding more time for patients. Three common approaches were identifiable in transcripts, those whom preferred pharmaceutical options, those whom preferred non-pharmaceutical therapies administered, in the first instance by themselves and those whom preferred to refer (usually to psychology).

GPs also detailed the fundamental importance of patient buy-in for dealing with depression as a health problem. When patients do not buy-in to techniques for dealing with depression, for example taking medication, it means that other agreed upon techniques should be drawn on. Likewise, if patients do not buy-in to having a health problem called depression, treating and managing the problem remains elusive. As the quotes from GPs in Table 5 show, recording diagnoses of mental health problems in the medical record was a highly sensitive and confidential matter.

Thus, coupled with the varied understandings of depression and depression work, there is still limited agreement and engagement with a shared set of techniques that deal with depression as a health problem in primary care. Figure 4 outlines the role that cognitive participation has for embedding a model of depression care. In addition to this, construct three collective action advocates that depression work requires agreement about how care is organised -- who is required to deliver care, and their structural and human interactions. The NPT concept of collective action is defined as purposive action aimed at a clear goal and is influenced by both organisational (external) factors and immediate (internal) factors. Collective action is explained as a combination of skill-set workability (how work is allocated and performed), interactional workability (how well work fits into current practice), relational integration (accountability and confidence within care network), and contextual integration (structures and procedures that facilitate the work).

There are a number of external and internal factors required to support and enable this construct. This includes the development of organisational policies about the practice team skill set that is required for depression care, and how the work is to be allocated to optimise these skill sets within available funding mechanisms. Table 5 shows that there needs to be considerable attention to how work is organised and allocated at the organisational level. Practice nurses, for example, expressed some doubts about their role in delivering depression care, as did receptionists. They felt their current role in depression care was to play a supportive, listening role to patients given the time constraints of GPs. But they also mentioned being uncertain about the information to give to patients; they expressed some feelings of being poorly equipped to deal with depression and suggested they would need specific training. Reception staff said that if they were to play a role in depression care it would be difficult for them to know if a patient was depressed. In turn, GPs were not certain which aspects of depression care could be allocated to practice nurses. While they also acknowledged the important role that the reception staff played, it was uncertain how work would be allocated at an organisational level.

In addition to internal skills, keeping abreast of policy and funding changes and having mechanisms in place to ensure that such policies are reviewed is a necessary part of organising depression work. External support from government and other relevant bodies is also needed to develop functional communication pathways within organisations (both between staff and with patients) and the technological systems, for example, messaging systems, electronic medical record support, newsletters, and emails to patients. Sharing of medical records within and beyond the organisation and issues of confidentiality need to be supported and discussed. The physical infrastructure of many primary care organisations needs to be improved for staff to meet regularly and conflict resolution mechanisms also need to be developed to actively address disagreements.

Without resourcing, formal agreements, and shared understanding it is not surprising that construct four, reflexive monitoring, remains underdeveloped in primary care organisations. Figure 4 shows this in relation to the additional three constructs. The fourth construct is based on the proposition that depression work requires the ongoing assessment of how depression care is done. Currently, the opportunities for primary care organisations to self-assess the effectiveness of their depression care are minimal.

One of the reasons for this is the lack of consistency regarding the recording of diagnostic information about depression or distress, particularly in electronic record systems. In almost every site, staff noted that they did not have a system in place for ensuring accurate records on diagnosis or treatment. No organisation could produce a list of people currently being treated for depression (either by a GP or via referral) other than those currently prescribed antidepressants. Most agreed that prescribing information was the only reliable information that they recorded in a systematic way for people with depression; yet they also stated that many patients were not using prescription drugs for the management of depression. The only other accurate recording of depression work was that obtained via the billing software for 'structured plans of action for mental health' that were charged.

The current primary care environment is limited in the extent to which a systematic approach to reflexive monitoring can be implemented without substantial improvement in organisational infrastructure or ongoing financial support. Reflexive monitoring was also seen to require a process for reviewing the communication pathways between GPs and others involved in depression care, particularly psychologists. There was a noted lack of communication and available processes between general practice and psychology.

At present, the schedule for patient follow-up and monitoring is individually tailored by the individual clinician and the patient concerned. There is no systematic way of ensuring that patients return for follow-up, or of checking on whether they have attended when referred. There is no agreement on how often follow-up visits should occur. Based on the results discussed above, a system of reflexive monitoring might also include a review of understanding of what constitutes depression and review of techniques for dealing with it as a health problem. As populations change, what is considered depression may shift, and the techniques available to identify and manage it will evolve. Ensuring that an organisation keeps up to date with these developments is an important part of the monitoring process.

## Discussion

Previous studies have identified that efforts to change organisational and professional practice are best preceded by the effort to understand what is already happening [26]. Without understanding the organisational structure and processes currently in place for depression care, implementing and embedding change from the outside will be of limited success. This is confirmed by the limited uptake of guidelines for depression management by GPs [35].

We have used data collected from six primary care organisations to develop a conceptual framework for implementing best practice depression care that is informed by NPT. This theoretical approach clearly demonstrates the existing normative and structural constraints in current depression care practice which will negatively impact upon the implementation of new models of depression care [30]. While this evidence has been generated from primary care organisations based in Australia, there are shared patterns with other international studies that have sought to implement change in primary care [24,26,27].

Our data confirm that change at the level of the practitioner through education interventions alone is unlikely to facilitate much required organisational and system-level change [36]. Current mental health reform has been focussed quite narrowly on the area we describe as 'collective action' with an emphasis on improving individual practitioner skill-set through targeted mental health education. While options have emerged to shift the allocation of work from GPs to other health care providers like psychologists, this has not been accompanied by adequate infrastructure to improve communication pathways and develop ways of working together.

Although educational interventions to change individual practice of doctors have shown limited success, practice nurses are set to play a key role in depression care within the primary care setting. To facilitate this role, training in mental health care tailored to the requirements of practice nurses is needed. The International Council of Nurses (ICN, 2008) position statement on mental health advocates for the integration of mental health into nursing curriculum at basic, post-basic, and continuing education levels. All professional nursing bodies need to ensure that adequate education is provided so that the confidence of practice nurses can be improved.

Training of professional staff needs to be combined with the development and implementation of information systems that can measure and report on mental health indicators and outcomes. These information systems require funding and maintenance and training of staff in how to use measures and report on these. The use of measurements for mental health is further complicated by the lack of agreement about the most appropriate tools for detection and measurement of depression within the primary care setting.

The processes of achieving change requires action across the spectrum of levels that moves from individuals, the group/team, organisation to larger system/environment [17]. This requires understanding and agreement about depression at all levels of organisational and systems change. Primary care organisations require adequate infrastructure from the basic level of having physical team meeting spaces available to the more complex level of information systems to support the work. Our findings suggest that even a simple intervention, like introducing routine meetings about the system required to support depression care, could have far reaching benefits. Such meetings would need to address the constraints that require urgent attention for the routine implementation of an effective model and system of depression care that relate to how depression and depression work is understood, its perceived legitimacy as a health problem, and the limited mechanisms for dealing with this diffuse phenomenon.

Our study participants held a different view of depression and depression work to the traditionally applied psychiatric viewpoint [37]. This suggests that without shared agreement about what primary care means by the term depression, diagnosing and developing adequate treatment and management pathways will remain difficult. Without agreement, people (staff and patients included) will not engage (buy-in) and cognitively participate in depression work and share in techniques to address the problem. There is a pressing need to better understand the way in which physical and emotional health is intertwined as part of this process.

Patients and community stakeholders consulted as part of our earlier re-order work have already indicated what they desire in an effective model of depression care [18]. Embedding such a model rests on all people knowing the division of labour and this being aligned with the individual and collective values of organisations. Data indicated that elements of an effective model of depression care existed in organisations; however, we found that this differed within each organisation and professional group. This has been identified in other research studies based in the UK to improve the quality of care for people with mental health [38]. To facilitate implementation, system level changes are required that enable people to meet, discuss, and share information either face to face or through electronic systems; all of which are difficult to achieve in fee-for-service reimbursement systems. For any activity to become a routine part of clinical work those undertaking it need to be convinced that the work are they doing is worth the effort. At present the normalised processes for reflexive monitoring of depression work is highly individualised, invisible, and unsystematic. There will need to be considerable effort put into how to monitor the effectiveness of any model and system of depression care. In doing so, primary mental health care needs to be recognised at as an operating systems in its own right [38].

The concept of reflexive monitoring that we present in this paper requires that practitioners have time to review reliable, routinely collected data about their delivery of depression care. The organisations in our study were a long way from being able to undertake this monitoring. Such monitoring requires time away from direct patient care to review the data, discuss the data, and devise strategies to make improvements. Currently, in a mainly fee-for-service environment, the funding mechanism to support this aspect of depression care is absent from the Australian primary care setting. Other suggested mechanisms from the organisational level for reflexive monitoring included Balint-style group meetings. Study participants identified that this would have the dual role of group monitoring of diagnosis and treatment of depression while providing an organised support mechanism for professional. This could assist in the development of a culture of continuous learning within organisations and if held as multidisciplinary. Balint group meetings could provide a mechanism for different professional groups to develop new languages and ways to communicate across disciplinary boundaries and divisions. This is likely to require a significant level of financial investment; particularly in information and communication technology. Moreover, adequate attention to all four constructs will be required not just the development of skill-sets.

## Strengths and limitations

A strength of the re-order study is the depth and detail generated about primary care as a CAS by utilising a number of quantitative and qualitative research methods. A limitation is our involvement of organisations from a practice based research network. Our findings should be interpreted with this is mind, and it may be that implementing change is even more complex in organisations not interested in research. Limitations also exist in primary care organisations around using electronic health record audit data beyond description; the input of data can be inconsistent and diagnostic categories used within practices and by individual GPs vary. Not all organisations achieved whole of practice staff commitment to the project. This has limited our ability to provide psychologist's views on their current understanding and practices for depression and the future organisation of care. Finally, as other studies have shown, the intensity of data collection for this research requires considerable resources and diversity in the research team.

## Conclusions

Ideas about what is required for an effective model and system of depression care in primary care need to be accompanied by theoretically informed frameworks that consider how these can be implemented. The conceptual framework we have presented has some obvious messages, but developing each of these constructs within the complexity of current organisational practice should not be underestimated. Currently, mental health reforms have been at the level of collective action with little attention to building organisational capacity for developing coherence and cognitive participation. Implementation of systems and practices for reflexive monitoring are some way off. The conceptual framework we have developed can be used beyond the organisational level to develop common language around each construct between policy makers, service users, professionals, and researchers. This shared understanding across groups is fundamental to the effective implementation of change in primary care for depression. Our next step is to develop an intervention around the use of our developed framework and to test this in a randomised controlled trial to determine the impact of contextually specific organised depression care on health outcomes.

## Competing interests

The authors declare that they have no competing interests.

## Authors' contributions

JG, CD, HH, FG, KH, and GB were responsible for the concept design of the re-order project. JG, VP, CD, HH, FG, KH, GB, CJ and RK all contributed to analysis and interpretation of data used within this paper. MP contributed to substantive data acquisition and analysis of the data for this work. VP designed structured activities for the meetings, was responsible for data collection and facilitation of the organisational meetings, and completed data analysis of transcripts with JG. HH, JG, VP, RK, MP, CJ, and a representative from the funding body (APHCRI) attended the final workshop to test the conceptual framework with study participants. JG developed the synthesis of the conceptual framework presented in this paper with intellectual contributions from all authors. All authors have read and approved this final version of the manuscript.
